# Relaxed Rotational and Scrunching Changes in P266L Mutant of T7 RNA Polymerase Reduce Short Abortive RNAs while Delaying Transition into Elongation

**DOI:** 10.1371/journal.pone.0091859

**Published:** 2014-03-20

**Authors:** Guo-Qing Tang, Divya Nandakumar, Rajiv P. Bandwar, Kyung Suk Lee, Rahul Roy, Taekjip Ha, Smita S. Patel

**Affiliations:** 1 Department of Biochemistry and Molecular Biology, Rutgers University, Robert Wood Johnson Medical School, Piscataway, New Jersey, United States of America; 2 Department of Physics and Center for the Physics of Living Cells, University of Illinois at Urbana-Champaign, Urbana, Illinois, United States of America; 3 Center for Biophysics and Computational Biology, University of Illinois at Urbana-Champaign, Urbana, Illinois, United States of America; 4 Howard Hughes Medical Institutes, Urbana, Illinois, United States of America; University of Iowa, United States of America

## Abstract

Abortive cycling is a universal feature of transcription initiation catalyzed by DNA-dependent RNA polymerases (RNAP). In bacteriophage T7 RNAP, mutation of proline 266 to leucine (P266L) in the C-linker region connecting the N-terminal promoter binding domain with the C-terminal catalytic domain drastically reduces short abortive products (4–7 nt) while marginally increasing long abortives (9–11 nt). Here we have investigated the transcription initiation pathway of P266L with the goal of understanding the mechanistic basis for short and long abortive synthesis. We show that the P266L mutation does not alter the affinity for the promoter, mildly affects promoter opening, and increases the +1/+2 GTP *K*_d_ by 2-fold. However, unlike wild-type T7 RNAP that undergoes stepwise rotation of the promoter binding domain and DNA scrunching during initial transcription, the P266L mutant does not undergo coupled rotational/scrunching movements until 7 nt RNA synthesis. The lack of rotation/scrunching correlates with greater stabilities of the initiation complexes of the P266L and decreased short abortive products. The results indicate that the increased flexibility in the C-linker due to P266L mutation enables T7 RNAP to absorb the stress from the growing RNA:DNA hybrid thereby decreasing short abortive products. Increased C-linker flexibility, however, has an adverse effect of delaying the transition into elongation by 1–2 nt, which gives rise to long abortive products. However, a mutation in the upstream promoter region greatly decreases long abortive products in P266L reactions, rendering the combination of P266L and A-15C promoter a desirable pair for efficient *in vitro* transcription for RNA production. We conclude that the conformational rigidity in the C-linker region conferred by the proline at position 266 is responsible for the undesirable short abortive products, but the rigidity is critical for efficient promoter clearance and transition into elongation.

## Introduction

All DNA-dependent RNA polymerases (RNAP) synthesize and release short RNA products during transcription initiation, but the exact mechanism or the rationale for abortive cycling synthesis observed also *in vivo* [1] is not understood. Bacteriophage T7 RNAP is a single-subunit enzyme widely used for *in vitro* synthesis of RNA polymers. It is a preferred enzyme for *in vitro* transcription, because of its simplicity, high specificity for its promoters, and no requirements for transcription factors. Similar to other RNAPs, T7 RNAP makes both short (2–8 nt) and long (9–12 nt) abortive products during transcription. A genetic screen identified P266L mutant of T7 RNAP as an enzyme that produces significantly reduced amounts of short (5–8 nt) abortive products [2]. Thus, P266L can serve as a superior enzyme for *in vitro* transcription reactions, except for the fact that it still makes long abortive products. In addition to its biotechnology applications, P266L is a valuable tool for exploring the mechanistic basis for abortive synthesis that can aid in the future design of better enzymes for *in vitro* transcription.

Being a single-subunit enzyme, T7 RNAP catalyzes all the stages of transcription from initiation to termination without relying on any transcription factors. The N-terminal domain in T7 RNAP serves as the promoter recognition domain during initiation and remains bound to the upstream promoter region throughout initiation. To accommodate the growing RNA:DNA hybrid during initiation, T7 RNAP undergoes step-wise promoter DNA scrunching and N-terminal domain rotation and refolding changes, which are well characterized by crystallography and Fluorescence Resonance Energy Transfer (FRET) experiments [3], [4]. The N-terminal domain releases the promoter after 8–12 nt RNA synthesis [5], which then triggers major conformational changes in the RNAP-DNA complex that results in the transition into elongation. At this time, the initial DNA bubble collapses and the subdomain H within the N-terminal domain refolds and undergoes a large movement to become part of the RNA channel for the final elongation complex [6]–[10]. Thus, rotation and refolding of the N-terminal domain allows this single subunit RNAP to catalyze both promoter-specific transcription initiation and promoter-independent elongation of RNA without requiring transcription factors.

**Figure 1:**
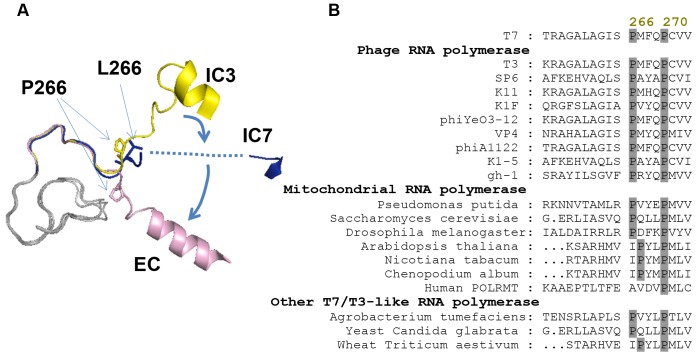
C-linker region of T7 RNAP. (A) The C-linker region (residues 251 to 296 in cartoon format) adopts different conformations in the initiation state (yellow, 1QLN, IC3), the elongation state (Pink, 1MSW, EC), and with 7 nt transcript bound (blue, 3E2E, IC7). The P266 and L266 residue is shown in stick format. The amino acids from 255 to 263 are disordered in the P266L structure (3E2E) and shown as dashed line. The direction of rotation of the linker near the hinge region is marked with arrows. The C-terminal domains (residues 300–883) of the three structures were aligned using Pymol (Molecular graphics systems). (B) Conservation of proline residue in the linker region between N-terminal domain and C-terminal domain at positions 266 and 270 in single-subunit RNAPs of phage, bacterium, and eukaryotic mitochondria. The N-terminal 1–300 amino acid sequence of T7 RNAP was used as a query in a BLAST amino acid search of the NCBI database for sequence alignment.

Proline 266 is present within the C-linker region that connects the N-terminal domain with the C-terminal domain near the pivot point of rotation (Fig. 1A). In the transition from the initiation complex with 3 nt RNA (IC3) to the elongation complex (EC), the bond between residues 267–268 is rotated by ∼180° and the helix from 248–256 is extended to residue 261 due to N-terminal domain rotation [6], [7]. Crystallography studies [4] were able to capture the P266L mutant in an initially transcribing complex with 7 nt RNA (IC7), but the C-linker region near L266 was disordered, which indicates that mutation of P266L confers flexibility to the C-linker region. Studies have shown that introducing additional prolines in the C-linker region inactivates T7 RNAP [11]. Thus, the specific conformation of the C-linker region is important for initial transcription and transition into elongation. Interestingly, P266 is conserved in T7/T3-like phage RNAPs and found in homologous mitochondrial/chloroplast RNAPs (Fig. 1B).

Earlier studies of P266L ascribed the higher processivity or reduced short abortive products during initial transcription to facilitated promoter release based on their observation that P266L has weaker affinity for the promoter [2]. However, a recent study reported that P266L binds the promoter with similar affinity as wild-type (WT), but has a delayed transition from initiation into elongation [12]. The authors proposed a hybrid-push model to explain the synthesis of short abortives in WT. Here, we have investigated the transcription initiation pathway of P266L to obtain better insights into the mechanistic basis for efficient initial transcription. By using a combination of biochemical and biophysical approaches, including limited proteolysis and FRET, we show that mutation of P266L mutation relaxes both the rotation and DNA scrunching changes for RNA lengths less than 7 nt, which stabilizes the short RNA:DNA hybrids, but delays promoter release, thus having discernible effects on short and long abortive synthesis.

## Materials and Methods

### Proteins

The P266L mutation was introduced into T7 gene 1 in plasmid pAR1219 (kindly provided by Alan Rosenberg and Bill Studier, Brookhaven National Laboratories) [13] using the single site-directed mutagenesis protocol (Stratagene). The sequences of mutagenesis primers were: Forward primer: 5′- GCG CTG GCT GGC ATC TCT CTG ATG TTC CAA CCT TGC G -3′, Reverse primer: 5′- CGC AAG GTT GGA ACA TCA GAG AGA TGC CAG CCA GCG C -3′. The mutant P266L T7 RNAP was overexpressed in *E. coli* strain BL21, purified following the method of Grodberg and Dunn [14] and found to be >95% pure by gel electrophoresis. The eluted enzyme was dialyzed in buffer containing 20 mM sodium phosphate (pH 7.7), 1 mM Na_3_-EDTA, 1 mM DTT, 100 mM NaCl and 50% (v/v) glycerol, and stored at −80°C. The concentration was calculated from its absorbance at 280 nm in 8 M urea using the molar extinction coefficient of 1.4×10^5^ M^−1^cm^−1^ [15]. WT T7 RNAP was purified by the same protocol as above.

### Oligonucleotides

Synthetic oligodeoxynucleotides (unmodified and 2-AP modified) with T7 promoter sequence were custom synthesized by Integrated DNA Technologies (Coralville, IA), gel-purified and quantified as previously described [16], [17]. The sequence of the non-template strand used in the steady state transcription assay is as follows: φ9: GCC GGG AAT TTA ATA CGA CTC ACT ATA GGG AGA CCT CAT CTT TGA A. φ9(A-15C): GCC GGG AAT TTA CTA CGA CTC ACT ATA GGG AGA CCT CAT CTT TGA A. Sequences of the non-template strand used in the walking experiments in the 5′ to 3′ direction and the fluorescence anisotropy experiments is the same as the ones used by Tang et al [5], [18]. The sequence of the DNA substrates used in the single molecular FRET experiments are as follows. The non-template strand sequence: 5′ – TGG CGA CGG CAG CGA GGC TAA ATT AAT ACG ACT CAC /*Cy3*-*C6-*T/AT AGG GAG AAC TAG ACG TTA TCA GCT TC – 3′. The template strand sequence: 5′ – GAA GCT GAT AAC G/*Cy5-C6*-T/C TAG TTC TCC CTA TAG TGA GTC GTA TTA ATT TA –3′. Duplex DNAs were prepared by mixing the template strand with the complementary non-template strand, heated to 95°C for 5 minutes and allowed to slowly cool to room temperature.

### Fluorescence anisotropy based titrations to measure the equilibrium dissociation constant

The equilibrium promoter DNA binding experiments were carried out at 25°C by fluorescence anisotropy measurements using 10 nM TAMRA-labeled DNA titrated with increasing concentrations of P266L T7 RNAP in buffer A (50 mM Tris-acetate, 50 mM sodium acetate, 10 mM magnesium acetate, 5 mM DTT) as described previously [18]. To obtain the dissociation constant (K_d_), the resulting fluorescence anisotropy vs. [P266L T7 RNAP] plot was fit to a quadratic equation as described [18].

### Extent of open complex formation

Fluorescence at 370 nm of 2-AP labeled promoter DNA at −4 template position (250–400 nM) and T7 RNAP (500 nM) mixed in 0.2 ml of buffer A was measured at 25°C upon excitation at 316 nm using a FluoroMax-2 spectrofluorometer (Jobin Yvon-Spex Instruments S.A., Inc.). Average fluorescence intensity was determined within the DNA concentration range after correcting for dilution and inner filter effect as described previously [19].

### Equilibrium K_d_ of initiating NTPs

A preformed complex of 2-AP labeled promoter DNA (250 nM) and T7 RNAP (500 nM) was titrated with increasing concentrations of 3′-dGTP (Trilink Biotechnologies, Inc). The change in 2-AP fluorescence was determined and corrected for dilution and inner filter effect. The fluorescence *F* versus 3′-dGTP concentration data were fit to the Hill equation (Equation 1) to obtain the average *K*_d_ of initiating 3′-dGTPs.
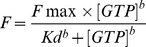
(1)where GTP represents 3′-dGTP, *F*_max_ is the observed amplitude and *b* is Hill coefficient.

### Steady state transcription assay

Transcription assays were carried out at room temperature (22–25°C) in 10 μL buffer A using RNase free water and in presence of an RNase inhibitor (44 units/mL RNasin® from Promega). A preincubted mixture of T7 RNAP and DNA was mixed with GTP (1 mM, final) spiked with [γ-^32^P]GTP (GE Healthcare/Amersham Biosciences), and other NTPs (0.5 mM each) to initiate the reactions. The reactions were quenched with EDTA (100–150 mM) after 60 s for the steady state transcription assay and 120 s for the RNA turnover assay. The RNA products were resolved on 23% polyacrylamide/4 M urea sequencing gels electrophoresed in 1.2× TBE buffer at 90–95 W (50°C). The gel was exposed to a phosphor screen; scanned on Typhoon 9410 PhosphorImager instrument (Molecular Dynamics), and the products were quantitated using the ImageQuaNT program (GE Healthcare).

### Trypsin digestion

Limited proteolysis of T7 RNAP (5 μM) by trypsin (2.5 μM, 8 units) was carried out at 25°C in buffer A, in absence or in complex with DNA (5 μM), and after walking the T7 RNAP-promoter complex using a combination of NTPs (GTP  = 1 mM, all other NTPs  = 0.5 mM each). Within 15 s of adding trypsin, the digested samples were loaded on a 4–20% Tris-glycine-SDS gel running at 50 V, and electrophoresed further at 100–150 V after the last sample was loaded. The protein fragments were visualized by Coomassie dye staining of the gel.

### Initial bubble collapse by stopped-flow 2-AP fluorescence measurements

Real-time measurements of 2-AP fluorescence changes from position −4 on the non-template strand during transcription reactions were conducted in the Kintek stopped-flow setup. A 370 nm long-pass filter was used to collect the emission fluorescence intensity of 2-AP fluorescence excited at 310 nm. WT/2AP-DNA or P266L/2AP-DNA (130 nM/100 nM) were rapidly mixed with limited NTP/3′dNTP mixtures (GTP  = 2 mM and other NTPs  = 0.8 mM) in the stopped flow setup. Fluorescence signals at each translocated position of transcription were averaged from 8∼10 shots.

### Single-molecule FRET

The fluorophores Cy3 and Cy5 are linked to −4T and +12T on the non-template and template strand, respectively. Biotinylated DNA duplexes were specifically bound to a PEG coated surface via a biotin-neutravidin link. The sample was imaged with 30 ms time resolution using an electron multiplying charge coupled device (CCD) camera (iXon DV 887-BI, Andor Technology, CT) and custom C++ [20] on a wide-field Total-Internal-Reflection (TIR) Microscope with a 532 nm diode laser (CrystaLaser, NV) for Cy3 excitation [21]. T7 RNAP (10 nM) was injected into a flow chamber with surface immobilized double-stranded DNA fragments as described previously [5]. After incubation for 5 min, the chamber was replenished with the same buffer without T7 RNAP to remove unbound T7 RNAP. Then, 1 mM GTP, CTP, ATP and 0.5 mM 3′-dUTP in the same buffer was introduced into the chamber, and the fluorescence was immediately monitored for 20 frames per imaging area, moving to different imaging areas after each measurement until the distribution reached steady state (∼35 min). The FRET efficiency *E*, was calculated using the equation 2 from apparent donor, *I_D_* and acceptor, *I_A_* signals after appropriate removal of donor and acceptor leakage and the background.
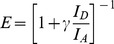
(2)where the most probable value of *γ*, the ratio of change in average acceptor intensity (*ΔI_A_*) to change in average donor intensity (*ΔI_D_*) before and after acceptor photobleaching [22], was calculated from 25 molecules after their acceptor undergoes photobleaching for each walking position of the T7 RNAP-DNA complex.

### Ensemble FRET measurements during initial transcription

Ensemble FRET measurements were carried out at 25°C on a FluoroMax-2 spectrofluorometer (Jobin Yvon-Spex Instruments S.A., Inc.) using buffer A. 100 nM dye labeled promoter DNA was mixed with 120 nM T7 RNAP followed by addition of limiting nucleotides to walk to appropriate positions (1 mM GTP, 0.5 mM other nucleotides). FRET efficiencies (E_FRET_) were determined by sensitized acceptor ((ratio)_A_) method described previously [23]. Mean D-A distances (R_da_) was calculated using equation 3 that relates the FRET efficiency, *E,* and the Förster radius (R_0_) of the dye pair. TAMRA-Alexa 647 dye pair were used for the promoter rotation assay (−22NT – TAMRA, N+5– Alexa 647) and Cy3– Cy5 dye pair were used for the scrunching assay (−4NT – Cy3, N+5– Cy5).
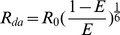
(3)R_0_ values for the TAMRA-Alexa 647 and Cy3-Cy5 dye pairs were determined to be 64 Å and 52 Å respectively, according to walking positions of T7 RNAP in transcription.

## Results

**Figure 2:**
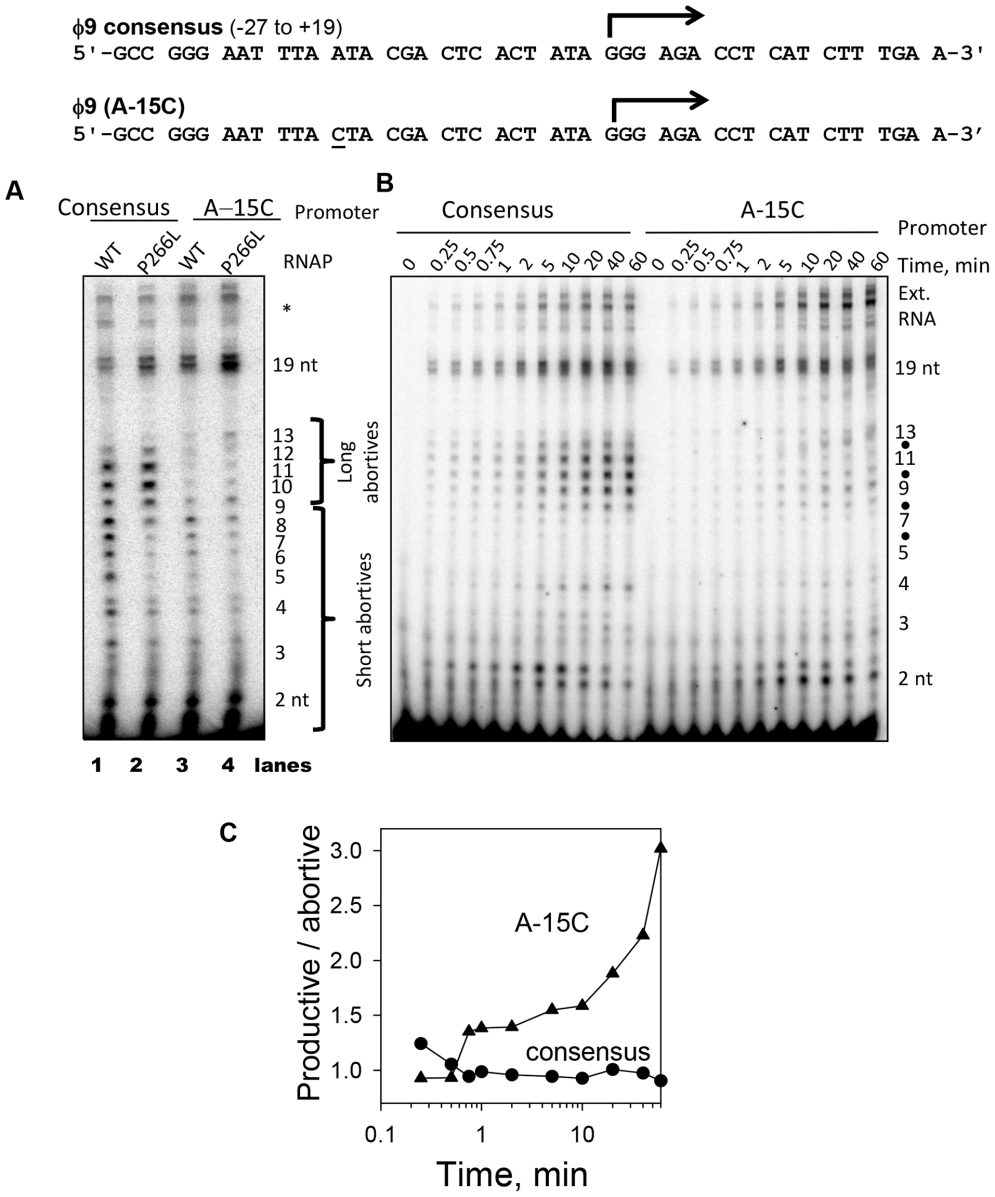
P266L mutation reduces short abortive without affecting the initial steps of transcription. (A) Transcription by WT or P266L T7 RNAP (5 µM) on the T7 φ9 consensus or φ9(A-15C) promoter (10 µM) was carried out at 25°C for 1 min and products were resolved on a 23% sequencing gel and visualized by [γ-^32^P]GTP labeling. (B) Time course of transcription by P266L(15 µM) on consensus and A-15C promoter (10 µM). (C) The productive (runoff) to abortive ratio is higher when the P266L T7 RNAP is used in combination with the A-15C promoter mutant than with the consensus promoter. The productive to abortive ratio was obtained from data in (B).

Transcription reactions were carried out on 46 bp synthetic promoters with consensus sequence from −27 to +19. On this promoter, the WT T7 RNAP makes the 19-mer runoff product and abortive products that can be broadly grouped into short (≤8 nt) and long (9 to 13 nt) in lengths (Fig. 2A, lane 1). It is clear from a comparative analysis that the P266L mutant of T7 RNAP makes significantly lower amounts of the short abortive products (Fig. 2A, lanes 1 and 2) but slightly higher amounts of the long 9–12 nt abortive products. These results are consistent with previous studies of this mutant [2], [12] and indicate distinct mechanisms for the formation of short and long abortive products.

### Long abortive products result from persistent promoter interactions

The long abortive products in WT T7 RNAP are produced mainly due to persistent promoter interactions [24]. Therefore, weakening the upstream promoter interactions by mutating the −15 AT in the consensus promoter to CG, result in lower amounts of long abortive products as well as efficient transition into elongation [24]–[26]. When tested on P266L, we find that A-15C promoter makes greatly reduced long abortives (Fig. 2A and 2B). P266L makes nearly 6-fold lower amount of long abortives on the A-15C promoter as compared to the consensus promoter. This indicates that similar to the WT T7 RNAP, the long abortives in P266L result from persistent upstream promoter interactions. The slightly higher amounts of long abortive products might suggest that P266L holds on to the promoter after 8 nt synthesis a little better than WT. Our results show that the combination of P266L mutation in T7 RNAP and A-15C mutation in the promoter reduces all abortives in the transcription reaction, resulting in a significantly high ratio of runoff to abortive products (Fig. 2C). Thus, we identify P266L and A-15C promoter combination as a preferred enzyme-promoter pair for *in vitro* RNA production.

### P266L mutation does not affect promoter binding, opening or initiating GTP binding

**Figure 3:**
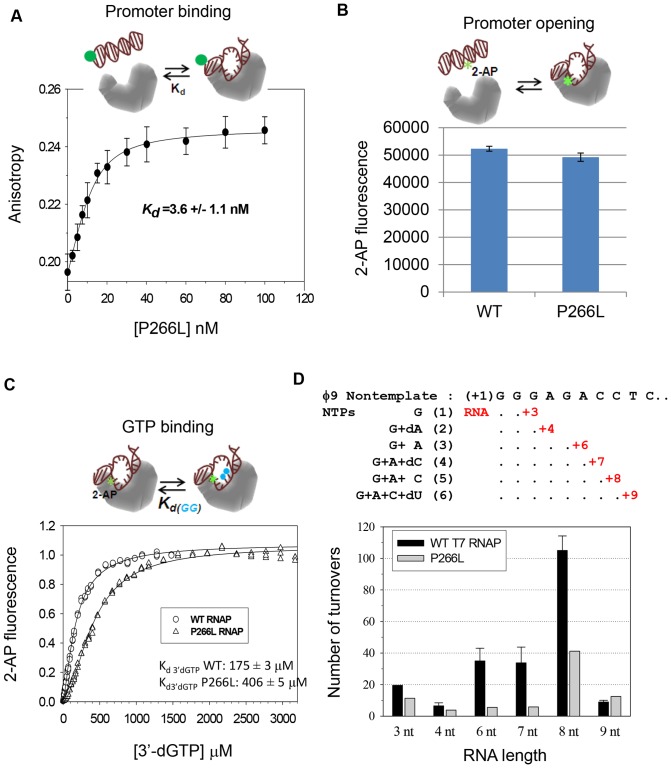
Promoter binding, opening, GTP binding and RNA turnover. (A) TAMRA fluorophore labeled promoter DNA (20 nM) was titrated with increasing P266L T7 RNAP (0 to 100 nM) and increase in fluorescence anisotropy was fit to the quadratic equation with *K*_d_ of 3.6±1.1 nM. Similar to the WT T7 RNAP, the P266L mutant did not cause significant changes in the TAMRA fluorescence intensity (<10%) upon binding. Effect of the intensity changes on the fitting was insignificant and corrected the same way as reported previously [23]. Shown here are averaged values with standard deviations (error bars) from multiple independent measurements, from which *K*_d_ was fitted. (B) The increase in 2-AP fluorescence at −4 in the template strand upon addition of WT and P266L indicate slightly lower promoter opening with P266L. The errors are standard deviation from 10–15 measurements. (C) Initiating GTP binding was monitored from fluorescence increase in the 2-AP (−4 position) labeled promoter DNA bound to T7 RNAP titrated with increasing 3′dGTP. The P266L binds to the initiating NTPs (3′dGTP) ∼2 times weaker than WT T7 RNAP (175 μM versus 405 μM) and Hill coefficients are 1.3±0.02 and 1.7±0.03 for WT and P266L, respectively. The errors represent the fitting uncertainty. (D) A complex of WT or P266L T7 RNAP (2 µM) and promoter DNA (1 µM) was mixed with limited NTPs or NTP and 3′-deoxy NTP mixture for 2 min at 25°C to allow RNA synthesis of the indicated lengths. The amount of RNA shown in µM is representative of the number of turnovers at each walked position for WT (black) and P266L (grey) T7 RNAP.

Our fluorescence anisotropy based DNA binding studies indicates that P266L has similar affinity for the consensus promoter as WT T7 RNAP before RNA synthesis. The equilibrium dissociation constant (*K*_d_) of ∼3–4 nM for the P266L-DNA complex (Fig. 3A) is similar to a 4 nM *K*_d_ of the WT T7 RNAP [18]. We find that P266L is slightly deficient in promoter melting, as measured by the increase in 2-aminopurine (2-AP) fluorescence at position −4 on the template (Fig. 3B) [23], [24], [27]. However, P266L does have a 2-fold weaker affinity for the initiating GTP analog, 3′-dGTP, as compared to the WT RNAP (Fig. 3C). The 3′-dGTP binds at +1 and +2 positions without reacting and provides the composite *K*_d_ of initiating GTPs [27]. Together our results indicate that P266L mutation in T7 RNAP does not alter the initial steps of promoter binding and opening and mildly affects +1/+2 NTP binding.

### P266L forms a stable initiation complex with 3–8 nt RNA transcripts

To determine if the decrease in the short abortive products is due to slower RNA dissociation rates in complexes with P266L as compared to WT, we measured the RNA turnover rate [10], [24]. Stably bound RNA transcripts will dissociate more slowly and hence accumulate in solution at slower steady-state or turnover rates. T7 RNAP complexes were walked to various positions using limiting NTPs/3′-dNTP and RNA turnover was measured after 3, 4, 6, 7, 8, and 9 nt synthesis. Our results show that P266L exhibits an overall slower turnover rates, we observed ∼2 fold reduced turnover rate after 3 nt and 4 nt RNA synthesis, 6-fold reduced turnover rate after 6 and 7 nt, 3-fold reduced turnover after 8 nt synthesis, but a slightly higher rate after 9 nt RNA synthesis (Fig. 3D). Thus, the results indicate that P266L forms more stable initiation complexes with 3–8 nt RNA transcripts as compared to WT. This also explains why P266L mutation enabled crystallization and structure determination of the intermediate initiation complexes bound to 7 and 8 nt RNA, which could not be accomplished with the WT T7 RNAP [4].

### Transition into elongation is delayed in the P266L mutant

Two papers have reported conflicting models of transcription by the P266L RNAP. Guillerez et al [2] proposed that the P266L mutant clears the promoter efficiently whereas a recent study has shown that transition into elongation is delayed by the P266L mutation [12]. To investigate the two models, we used several approaches including subdomain H refolding by trypsin proteolysis, the kinetics of initial bubble collapse using 2-aminopurine (2-AP) labeled DNA, and single molecule FRET to measure the efficiency of transition into elongation [5], [24], [28].

**Figure 4:**
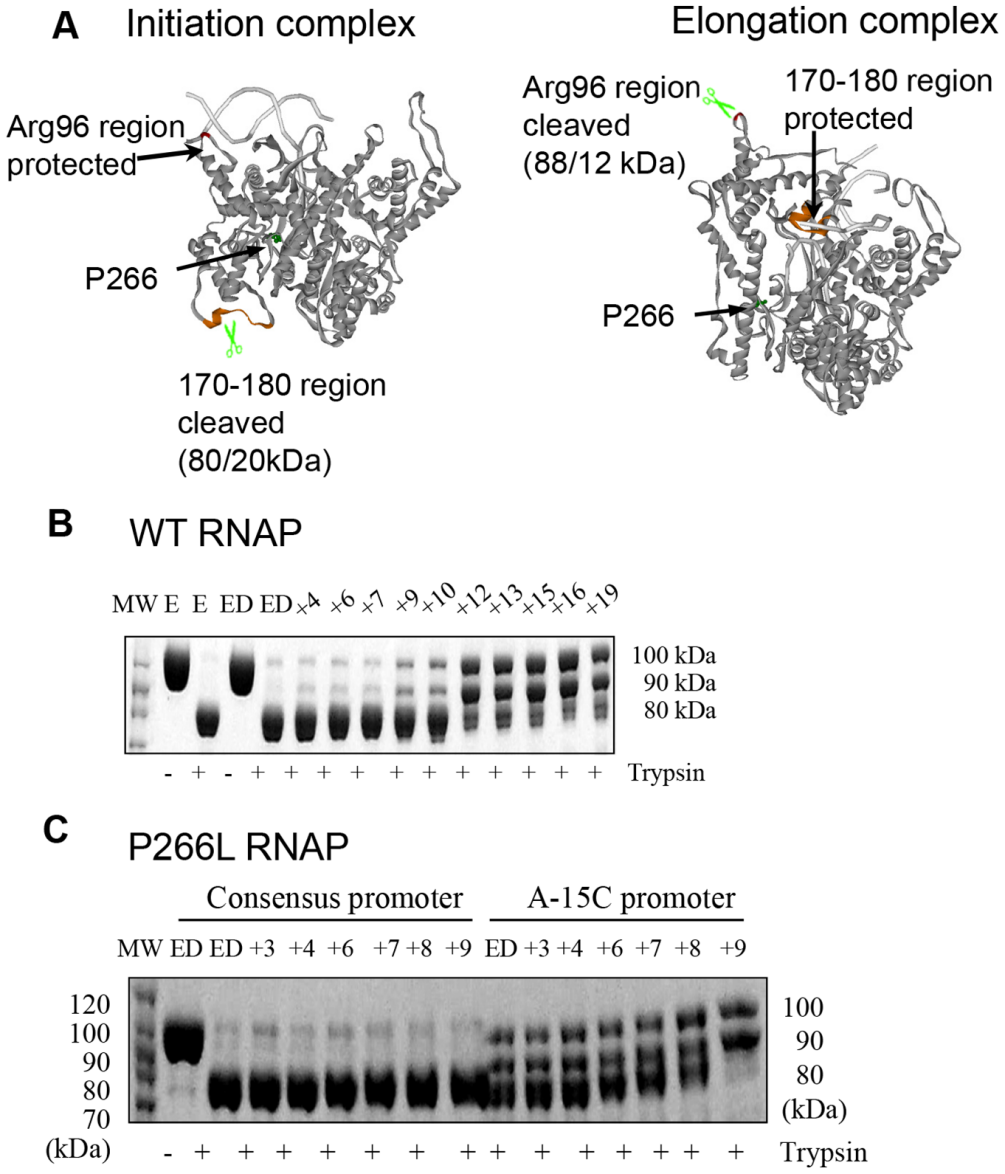
Subdomain H refolding is delayed in the P266L mutant. (A) T7 RNAP in initiation and elongation conformations have different exposure of Arg 96 (red color) and residues 170–180 (brown color) to trypsin digestion. Formation of an elongation complex is characterized by the disappearance of the 80 kDa fragment and the appearance of the 88 kDa fragment. P266 is colored in green. Limited trypsin digestion (15 s) of WT T7 RNAP after walking to +4 to +19 positions. E- RNAP, ED- RNAP:promoter complex (C) Same experiment as in panel B was carried out with P266L and consensus or A-15C promoter. The digestion pattern with the A-15C promoter shows that transition to EC occurs at +9 in the A-15C promoter, but not with the consensus promoter.

When T7 RNAP makes the transition into elongation, the 170–180 region in the N-terminal domain undergoes a large movement/refolding that allows the N-terminal domain to become part of the RNA exit channel [6]–[8]. In the initiation conformation, the 170–180 region is susceptible to trypsin cleavage yielding 80 kDa and 20 kDa fragments from digestion of the ∼100 kDa T7 RNAP [28]–[30]. In the elongation conformation, the 170–180 region is protected and hence the 80 kDa band disappears. When T7 RNAP releases the promoter, the AT-rich loop becomes trypsin sensitive at Arg 96 yielding an 88 kDa product (Fig. 4A) [24]. The trypsin cleavage patterns of the WT and P266L T7 RNAP were monitored after halting RNA synthesis from 4 nt to 19 nt. In WT, the 80 kDa band disappears and the 88 kDa band appears after 12 nt synthesis (Fig. 4B). On the other hand, P266L showed substantial amount of the 80-kDa band remaining even after 13 nt synthesis (Fig. 4C). These results indicate that subdomain H refolding is delayed in the P266L mutant. With the A-15C promoter, P266L undergoes efficient transition after 9 nt synthesis (Fig. 4C), similar to the WT [24]. These results indicate that transition into elongation is delayed in P266L on the consensus promoter and this correlates with inefficient promoter release and longer abortive RNAs.

**Figure 5:**
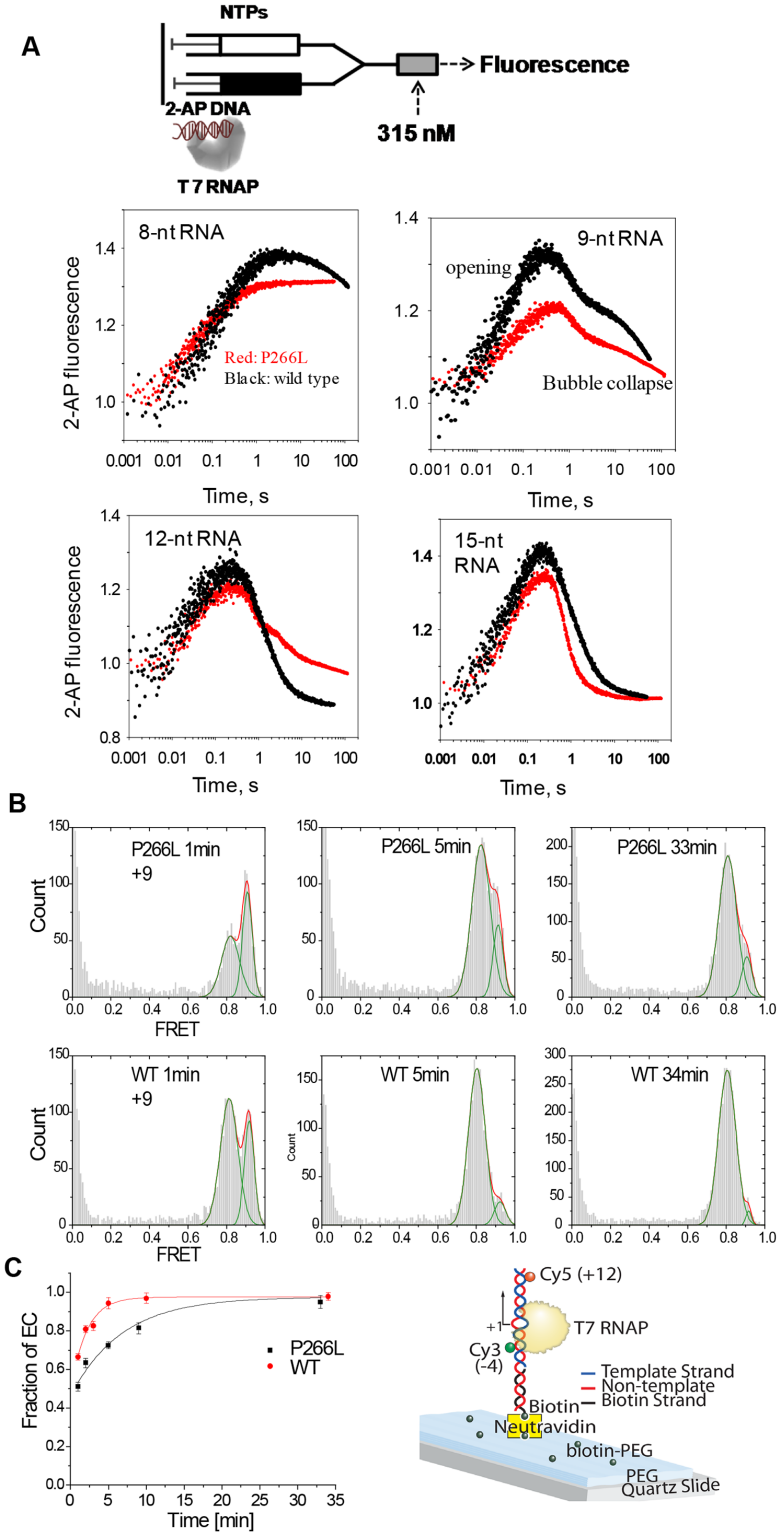
Upstream bubble collapse and transition into elongation are delayed in the P266L mutant. (A) Real time 2AP fluorescence monitors upstream bubble collapse. Representative time courses of 2-AP fluorescence changes from position −4NT in individual walking experiments (+8, +9, +12, and +15) were observed in the stopped-flow setup. The initial increase indicates rapid opening of the promoter and the decrease indicates bubble collapse. (B) Single molecule FRET histograms measure the rate of promoter unbending by WT and P266L T7 RNAP at +9. The number of DNA molecules that were analyzed to draw the smFRET histograms are as follows: P266L: 3110 at 1 min; 4164 at 5 min; 4883 at 33 min. WT: 3444 at 1 min; 3432 at 5 min; 5855 at 34 min. The *x*-axis shows corrected FRET (equation 2) and the *y-*axis represents the frequency of transcription complexes with the respective FRET values. Low FRET is observed in the elongation complex (EC) and high FRET is observed in the initiation complexes (IC). An increase in the low FRET population is observed over time after stalling at +9 using GTP+ATP+CTP+3′dUTP. Concentration of T7 RNAP-DNA, GTP, and NTPs was 10 nM, 1 mM, and 500 µM, respectively. (C) The fraction of EC versus time was fit to a single exponential function. The error bars in Fig 5C is the standard error from fitting smFRET histogram to a single exponential function. The WT (red circles) transitions to EC faster than P266L (black circles) at position +9 (τ = 4.5±1 min for P266L and 1.7±0.3 min for WT). The cartoon shows the layout of the smFRET experiments.

Upstream DNA bubble collapse is an essential step for the transition into elongation. The re-closure of the initially melted DNA region from −4 to −1 can be measured in real time by following the fluorescence intensity of 2-AP at −4 template position using the stopped-flow approach [5], [24]. Transcription was initiated by mixing a pre-incubated solution of 2-AP labeled promoter and T7 RNAP with limiting NTPs and 3′-dNTPs to halt RNAP at specific positions (Fig. 5A). A rapid initial increase in 2-AP fluorescence was observed due to open complex formation, but the subsequent decrease in 2-AP fluorescence is due to initial bubble collapse, which begins to occur at a slow rate after 8 nt synthesis in WT and after 9 nt synthesis in P266L. This indicates a 1 nt delay in the commencement of upstream bubble collapse in P266L. However, even after 12 nt synthesis, the transition into elongation is inefficient in P266L. Single molecule FRET experiments [5] using consensus promoter labeled with donor (Cy3) and acceptor (Cy5) at −4 and +12 positions, respectively, confirmed a slower conformational change after 9 nt RNA synthesis by P266L as compared to WT (τ = 4.5±1 min for P266L and 1.7±0.3 min for WT) (Fig. 5B). The initiation complex (IC) has a higher FRET because the DNA is severely bent and transition into elongation results in unbending of the DNA and a decrease in FRET.

### P266L mutation modifies promoter rotational movements during 4–6 nt RNA synthesis

**Figure 6:**
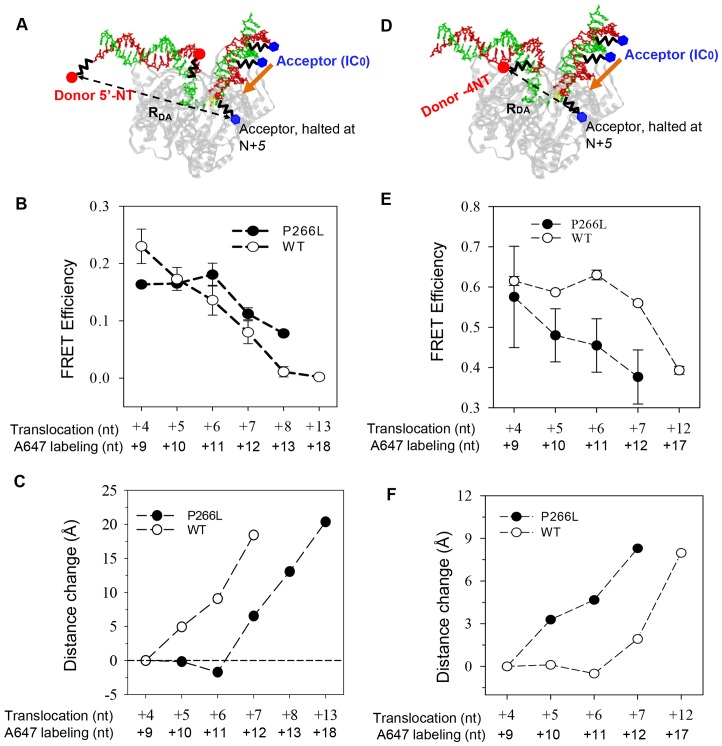
The P266L mutation modifies both rotation and DNA scrunching changes during initiation. (A) Cartoon illustration of FRET experiments to measure promoter rotation. The polymerase is in gray, the non-template strand in red and the template in green. Fluorescent donor TAMRA (red sphere) was introduced at position −22 in the non-template strand and acceptor Alexa 647 (blue square) at designated downstream positions on the template strand. Transcription complexes were walked to position *N* (+4 to +13) and FRET efficiency between donor (D) at −22 and acceptor (A) at *N*+*5* was measured to obtain the D-A distances (R_DA_, discontinuous line). (B and C) Average FRET efficiency and changes in D-A spatial distances are shown for P266L and WT T7 RNAP (D) Cartoon illustration of FRET experiments to measure DNA scrunching. The donor Cy3 (red sphere) was labeled on position −4 and acceptor Cy5 (blue square) labeled on downstream *N*+*5* positions as above. (E and F) Average FRET efficiency and changes in averaged D-A spatial distances between Cy3 at the upstream edge (−4) and Cy5 at the downstream *N*+*5* positions with the P266L and WT T7 RNAP complexes. The error bars of FRET efficiency represent the standard deviations from multiple independent measurements (N≥3).

Previous studies using FRET measurements have shown that the growing RNA:DNA hybrid is accommodated by T7 RNAP through gradual promoter rotation and DNA scrunching changes [3]. It has been proposed that DNA scrunching and/or rotational changes can result in “stressed” initial complexes that can push back on the RNA:DNA hybrid to release the RNA products in abortive cycling to relieve the stress [12], [31], [32]. To determine if P266L mutation changes the rotational and scrunching mechanism to alter the stability of the RNA:DNA hybrids during initiation, we probed these steps directly using our previously developed FRET methods [3]. A fluorescent donor (D) (TAMRA) was incorporated at upstream −22 position on the promoter and an acceptor (A) Alexa Fluor 647 (A647) was positioned on the template at *N*+5 position, where *N* is the length of RNA transcript (Fig. 6A). The upstream promoter is bound by the N-terminal domain and its step-wise rotation away from the active site causes an increase in D-A distance as the RNA:DNA hybrid grows during initial transcription [3], [4]. Thus, in the WT reactions, the observed FRET progressively decreases or the D-A distance progressively increases as the RNA grows from 4 and 8 nt (Fig. 6B and 6C). Interestingly, the same measurements with the P266L mutant showed that the inter-dye distances remain constant between 4 and 6 nt synthesis. After 7 nt synthesis, there is a sudden ∼10 Å increase in distance, followed by another ∼5 Å increase after 8 nt synthesis (Fig. 6B and 6C). This indicates that P266L accommodates the 4–6 bp long RNA:DNA hybrid without undergoing the same promoter rotational changes as observed in the WT T7 RNAP. The rotation measured after 7 and 8 nt RNA is consistent with the rotation observed in the crystal structures of P266L ternary complex with 7 and 8 nt RNA [4]. The results indicate that the rotational changes in T7 RNAP during initial transcription are altered due to the P266L mutation, which correlates with the more stable ternary complexes with the 4–6 nt long RNA transcript.

### P266L mutation modifies DNA scrunching during 4–6 nt RNA synthesis

**Figure 7:**
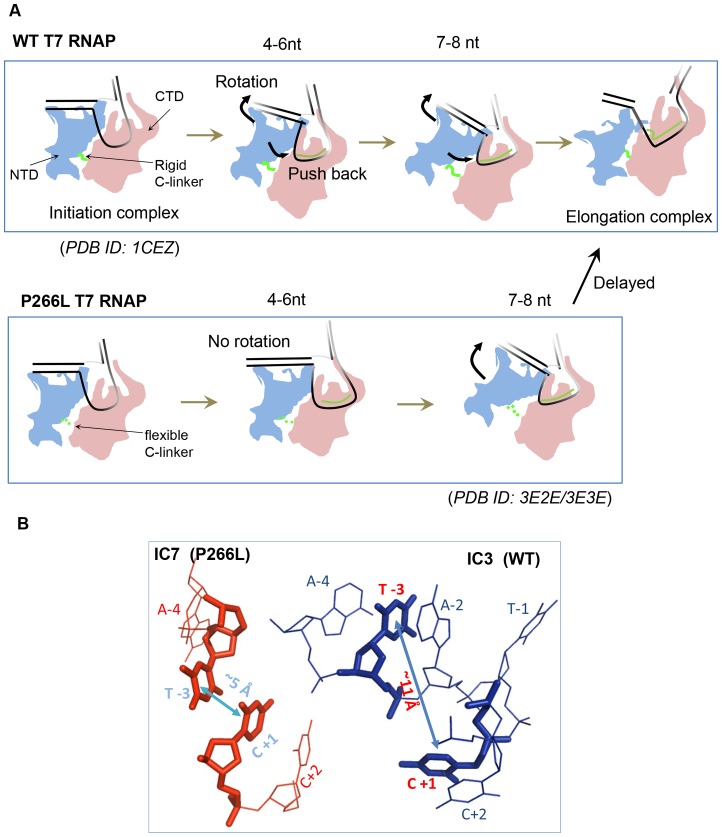
Distinct transcription initiation pathways of WT and P266L T7 RNAP. (A) The transcription initiation pathways of WT T7 RNAP (top) and P266L T7 RNAP (bottom) are shown in cartoon format to illustrate the distinct intermediate conformations. The N-terminal domain is shown in blue, C terminal domain in red, DNA in black and RNA in green. Movement of the N terminal domain is marked by the arrow. Both WT and P266L T7 RNAP bind, bend, and open the promoter DNA from −4 to +2 to the same extent. The rigid C-linker of WT favors progressive rotation of the upstream end of the promoter to accommodate the growing hybrid from +4 to +6 positions, which pushes against the N-terminal domain driving the rotation of the promoter. The pushback from the N-terminal domain destabilizes the RNA:DNA hybrid and leads to abortive synthesis in WT. The flexible C-linker of P266L mutant (bottom panel) accommodates RNA extension up to 6 nt without significant promoter/N-terminal domain rotation. The reduced DNA scrunching together with the absence of promoter rotation in the 4–6 nt RNA range in P266L suggests that the growing hybrid is accommodated by an alternative pathway. After 6 nt RNA synthesis, promoter rotation and scrunching resumes in P266L. The weakened promoter interactions in WT after 9 nt RNA synthesis allow release of the N-terminal domain and transition into elongation. Persistent promoter interactions delay the transition in P266L. (B) Template strand scrunching in P266L RNAP with 7 bp RNA:DNA. Template strand from the IC3 (PDB: 1QLN) and IC7 (PDB: 3E2E) crystal structures showing decrease in the distance between C+1 and T-3 in the IC7 structure (brown) compared to the IC3 structure (blue).

Another conformational change that occurs during initial transcription is DNA scrunching. T7 RNAP scrunches the template DNA while undergoing rotation to accommodate the growing RNA:DNA hybrid during initial transcription [3]. FRET measurements between the upstream edge of the initial transcription bubble (−4 position in the non-template strand) and *N+5* position in the template strand representing the downstream edge of the growing bubble, measures DNA scrunching (Fig. 6D). The WT T7 RNAP shows minimal changes in the inter-dye distance between the upstream and downstream edge of the initial bubble as RNA length increases form 4 and 6 nt (Fig. 6E and 6F). In contrast, P266L shows a progressive increase in the inter-dye distances as RNA length increases from 4 and 7 nt; 3–4 Å increase by 6 nt RNA synthesis and additional 5 Å after 7 nt synthesis (Fig. 6E and 6F). These results indicate that there is less scrunching during synthesis of short (4 to 6 nt) RNA transcripts in P266L as compared to WT T7 RNAP. After 7 nt synthesis, however, P266L follows the same rotational/scrunching pathway as WT T7 RNAP. The scrunching after 7 nt synthesis is consistent with the crystal structure of P266L with 7 nt RNA where the distance between T-3 and C+1 is ∼5 Å and hence shorter than the distance of ∼11 Å in the unscrunched ternary complex with 3 nt RNA (Fig. 7B). The combined measurements of promoter rotation and scrunching suggest an altered transcriptional initiation pathway of the P266L, in which there is minimal promoter rotation and scrunching changes between 4–6 nt RNA synthesis.

## Discussion

The P266L mutation was originally identified by genetic selection in *E. coli* as part of an *in vivo* screening effort for identifying T7 RNAP mutants with increased initial processivity [2]. Proline 266 is conserved in T7/T3-like phage RNAPs and also found in homologous mitochondrial/chloroplast RNAPs (Fig. 1B). *In vitro* transcription assays showed that purified P266L produced lower amounts of short abortive products, but curiously, longer abortive products (11–13 nt) were still produced [2]. While the mechanism was not determined, weaker promoter binding affinity, earlier promoter clearance, and/or additional flexibility of the polymerase in the C-linker region by P266L mutation were proposed as factors for selective reduction of shorter abortive products. Recent studies [12] of the P266L RNAP reported no change in promoter binding affinity of the P266L and a delayed transition into elongation, which contradicts previous studies [2]. Our biochemical characterization of the P266L are in agreement with Ramirez-Tapia and Martin [12] and shows that P266L has similar promoter affinity as WT, although it is slightly deficient in melting the promoter and has about 2-fold weaker affinity for the initiating nucleotides. We also show that P266L forms more stable initiation complexes with 4–8 nt RNAs that dissociate more slowly as compared to WT T7 RNAP. Because P266 is located away from the promoter binding site and the active site, it is unlikely that the P266L mutation directly stabilizes the RNA:DNA hybrid to selectively reduce shorter abortive transcripts. More likely the effects are allosteric and result from increased local flexibility of the C-linker when P266 is changed to L. Proline has an exceptionally rigid conformation compared to other amino acids and often plays a critical role in protein folding pathways. The importance of some flexibility in the C-linker was demonstrated when mutation of other amino acids in the C-linker to proline (G259P, A260P, G263P) inactivated promoter specific initiation [11]. P266 is present near the hinge region of the C-linker (residues 258–266) connecting the C-terminal domain to the N-terminal domain that undergoes most changes during initial transcription.

Previous FRET studies of T7 RNAP have shown step-wise rotation of the N-terminal domain during 5–7 nt RNA synthesis in a direction away from the active site to make room for the elongating RNA:DNA hybrid and to avoid steric clashes between the nascent RNA and the thumb domain (residues 370–410) [3]. Concurrently, downstream DNA in the initial bubble region is compacted in the active site (DNA scrunching). It was proposed that the tendency to reverse scrunching and rotation during initial transcription could be a mechanism for abortive synthesis [3]. Recently from studies of P266L, Ramirez-Tapia and Martin [12] have proposed a similar mechanism of abortive synthesis where they suggest that the push back from the rotated N-terminal domain destabilizes the RNA:DNA hybrid to produce the short abortive products. In their model, however, DNA scrunching does not play a major role in abortive synthesis. The reversal or push back model that explains abortive synthesis in WT T7 RNAP is illustrated in Fig. 7A. To determine if P266L follows the same pathway, we measured the rotational and scrunching changes in P266L at each stage of RNA synthesis from 4 to 12/13 nt using ensemble FRET methods, which were previously used to characterize such changes in the WT RNAP [3]. The increased flexibility of the C-linker region, due to the P266L change, could decrease the push back on the RNA:DNA hybrid to explain the lower amounts of abortive products.

Our ensemble FRET studies show that P266L undergoes minimal rotation during its processive stage of 4–6 nt RNA synthesis. The rotational movement does not appear in P266L until the synthesis of 7 nt RNA, which is ∼2 nt later than the commencement of rotational movement in WT T7 RNAP. Similarly, there is little DNA scrunching during the synthesis of 4 to 6 nt RNA in P266L as compared to WT. The changes observed at 7 nt synthesis by FRET changes are consistent with the latest crystal structures of T7 RNAP with 7 and 8 nt RNAs [4]. In these structures, there is about 40° rotation of the upstream promoter and interacting N-terminal subdomains. Although DNA scrunching was not mentioned, close inspection of the crystal structure (Fig. 7B) with 7 nt RNA (IC7) shows that C+1 and T-3 on the template strand are separated by ∼5 Å compared to ∼11 Å in the complex with 3 nt RNA (IC3).

How does P266L delay the rotation/scrunching changes when faced with the same problem of nascent RNA clashing with the N-terminal domain? FRET data suggests that P266L uses an altered initiation pathway that does not involve rotation/scrunching until 7 nt synthesis (Fig. 7A). This alternative mechanism results in more stable initiation complexes with 4–8 nt RNA as compared to WT that undergoes rotation/scrunching starting at 4 nt RNA synthesis. Although the exact changes that enable P266L to accommodate the 6 bp hybrid are not known, it is possible that the flexible C-linker enables translational motions to remove the clash with the N-terminal domain. These motions however reach a limit and therefore after 6 nt synthesis, the growing RNA:DNA is accommodated by rotational/scrunching changes in P266L. Thus, the flexibility in the C-linker alters the mechanism of initial transcription that produces significantly less short abortive products, but the deleterious effect of delaying the rotational/scrunching changes is inefficient final transition into elongation.

Although previous studies of P266L suggested early promoter clearance [2], our results are in agreement with Ramirez-Tapia and Martin [12] showing delayed transition into elongation. We demonstrated delayed transition into elongation by a number of assays including subdomain H refolding by trypsin proteolysis, the kinetics of initial bubble collapse using 2-aminopurine labeled DNA, and single molecule FRET. How do delayed rotational/scrunching changes in P266L delay the transition into elongation? Reversal of rotational/scrunching conformational changes during initial transcription that produce abortive products can also aid the transition into elongation by enabling promoter release. When the RNA:DNA hybrid is <7 bp, the reversal or push back from rotational/scrunching dissociates the RNA, but when the RNA:DNA is >7 bp, the longer hybrid remains stable and the reversal or push back instead breaks the interactions with the upstream promoter region. In P266L, due to the flexible C-linker, either there is not enough stress buildup after 7–9 nt synthesis or the C-linker is unable to effectively transmit the stress from the rotational/scrunching changes to trigger the final release of the promoter after 9 nt synthesis. Therefore, the conformational rigidity conferred by P266 in the hinge region, which is responsible for the rotational/scrunching changes during initial transcription, is also important for timely and efficient transition into elongation. Interestingly, A-15C change in the upstream promoter region enables P266L to efficiently transition into elongation after 9 nt synthesis. The combination of P266L and A-15C promoter yields an overall increase in the runoff to abortive synthesis, as it is able to maintain processive transcription during early initiation and efficiently clear the promoter after 9 nt synthesis, indicating the utility of this pair in RNA production by *in vitro* transcription.
